# Axial compressive behavior of lithium slag and rubber concrete-filled steel tube stub columns

**DOI:** 10.1371/journal.pone.0318617

**Published:** 2025-03-18

**Authors:** Ying Yang, Jiongfeng Liang, Wanjie Zou, Caisen Wang, Jicheng Liu

**Affiliations:** 1 Faculty of Civil & Architecture Engineering, East China University of Technology, Nanchang, China; 2 College of Civil and Architecture Engineering, Guangxi University of Science and Technology, Liuzhou, China; 3 College of Architecture and Civil Engineering, Beijing University of Technology, Beijing, China; 4 College of Civil Engineering and Architecture, Wenzhou University, Wenzhou, China; 5 Key Laboratory of Engineering and Technology for Soft Soil Foundation and Tideland Reclamation of Zhejiang Province, Wenzhou, China; SASTRA Deemed University, INDIA

## Abstract

In order to solve the harm of industrial waste such as lithium slag and waste rubber to the environment, this paper put forward the structure of lithium slag and rubber concrete-filled steel tube (LSRuCFST) stub column, which was replaced cement and sand in concrete by lithium slag and waste rubber respectively. Through the axial compression test of 11 LSRuCFST specimens with various lithium slag replacement ratios (0, 10%, 20%, 30%) and rubber replacement ratios (0, 5%, 10%, 20%, 30%), the damage patterns and mechanical characteristics of LSRuCFST columns were studied. The experimental phenomena showed that the damage patterns of 11 specimens were similar to the ordinary concrete-filled steel tube (CFST) colunms. With 20% replacement ratio of lithium slag and 10% replacement ratio of rubber, the largest increase of bearing capacity was optimal. Then, the bearing capacities of LSRuCFST columns were investigated by domestic and international standards. Due to conservative predicted results, a modified computational formal for the LSRuCFST column bearing capacity was presented and verified by the test results of references.

## 1. Introduction

As the industry improves by leaps and bounds, huge amounts of industrial wastes and tailings are produced every year in China, which has become a major difficulty to solve. Lithium slag is one of the industrial wastes. In addition, due to the rapid development of transportation industries such as automobiles, the generation of waste tires worldwide is also increasing over time. Billions of waste tires are waiting to be processed every year. Therefore, it is imminent for the recycling of lithium slag and waste tires. Whereas supplementary cementitious materials [1–2] and granite waste [3–4] are used to make concrete, one of ways to reuse lithium slag and rubber particles is to replace some of cement and sand in concrete, which can not only deal with the problems of land occupation and environmental pollution, but also save resources. To a certain extent, the important properties of cement-based materials are improved, which has good economic and environmental benefits.

In order to study the carbonation resistance and compressive strength of lithium slag concrete, Ding et al. [5] designed different lithium slag content to partially replace cement, and an accelerated carbonation test of lithium slag concrete was carried out according to the standard method. The experimental results showed that when the lithium slag replacement ratio was 20%, the strength of concrete was the largest; when the replacement ratio of lithium slag was 40%, the range of concrete pore carbonation depth was the largest. Qi et al. [6] experimental studies showed that partial replacement of lithium slag for fine aggregate could enhance effectively the desiccation resistance, wear resistance, and chloride resistance of concrete. When the content of lithium slag exceeded 20%, the wear resistance of concrete would decrease. He et al. [7] realized it was feasible that lithium slag was used to prepare the ultra-high-performance concrete, and found that due to the performance characteristics of lithium slag, the hydration degree of ultra-high strength concrete would be improved and its elastic modulus would be increased. Qin et al. [8] replaced natural pebbles and cement with recycled coarse aggregate and lithium slag according to different replacement ratios for concrete configuration. According to the experimental results, the optimal mix ratio of recycled coarse aggregate and lithium slag was obtained, and in this case, the splitting tensile strength and bending strength of concrete were the best.

To study the effect of different rubber contents and rubber types on the strength and deformation of the concrete, Zheng et al. [9] found experimentally that the elastic modulus and compressive strength of rubberized concrete showed a decreasing trend with the growth of rubber content. Meanwhile, it also showed that the rubberized concrete had good ductility. For studying the wear resistance of rubberized concrete, Kang et al. [10] prepared concrete with silica fume and rubber particles to partially replace sand for wear tests, respectively. The contrast tests showed that the compressive strength of rubberized concrete would be reduced, but the wear resistance could be raised to a certain extent. Xue et al. [11] investigated and found that the rubberized concrete as a new material had the effect of improving dynamic performance of building structures and reducing seismic response of those. Liu et al. [12] proposed the fatigue equation of rubberized concrete through test data, and predicted the fatigue performance of concrete for different rubber particle content. In addition, the toughness of the concrete raised with the growth of rubber particle content. For making full use of the characteristics of both rubber and fiber reinforced polymers, Youssf et al. [13] showed that the rubberized concrete with FRP not only has not been reduced in compressive strength, but also retained the good performance of ductility enhancement, and at the same time, due to the rubber admixture can significantly enhance the impact resistance of concrete, which indicated that it had good seismic performance. Due to the hydrophobicity and high deformability of rubber materials, the binding capacity of rubber powder and cement stone was poor. Gerges et al. [14] have shown that adding the rubber powder to the concrete reduced the concrete compressive strength, but could improve the toughness, and impact resistance of the concrete. Yang et al. [15] investigated the influence of the rubber particle size, the rubber content, and the thickness of the steel tube on the performance of circular steel tube confined rubberized concrete (STC-RuC). By analyzing the experimental results, one could conclude that adding rubber particles into concrete could reduce the density of concrete. The stiffness and ultimate strength of STC-RuC decreased with the rubber replacement ratio, but increased with the rubber particle size. And in the meantime, it could be concluded that the ductility and energy dissipation increased with the growth of rubber content. A. et al. [16] analyzed the effect of active confinement and rubber content on the strain and strength of the concrete. The results of the experiment demonstrated that the growth of the rubber content led to decreasing the compressive strength of concrete. However, the axial and lateral deformation increased with the content. This also implied that the ductility coefficient of the concrete increased with the content.

In a word, the conclusions of domestic and foreign researchers had obtained the consensus of scholars, namely, with the growth of lithium slag content, the compressive strength of lithium slag concrete increased first and then decreased; As the incorporation of rubber particles increased, the bearing capacity of the rubberized concrete-filled steel tube column specimens showed a decreasing trend, but it could raise the ductility of the structure. In order to make full use of industrial waste and reduce the use of cement and sand, which can reduce CO_2_ emissions and improve the mechanical properties of CFSTs, this paper studied the axial compression properties of concrete-filled circular steel tubular stub columns with lithium slag replacement ratios and rubber replacement ratios as control parameters, which provided a reference for the design, production, and application of such structures.

## 2. Experimental program

### 2.1 Experimental materials

**Fig 1:**
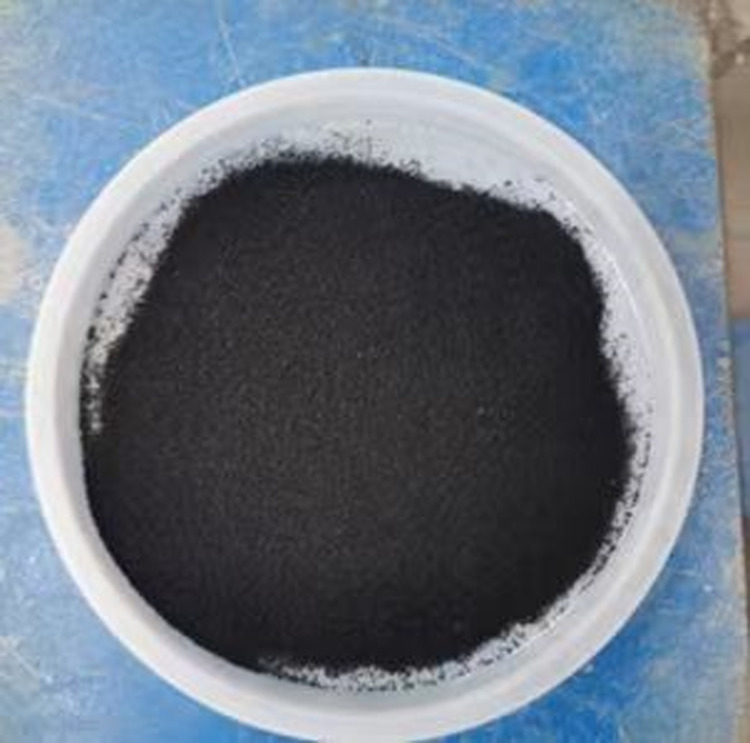
Rubber particles.

**Fig 2:**
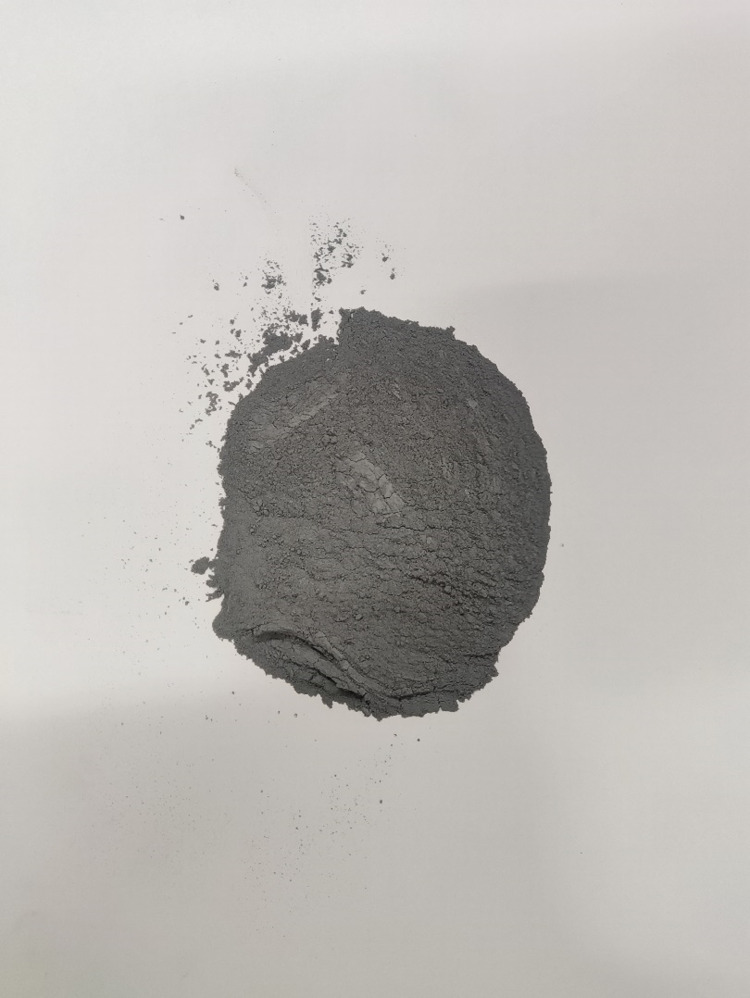
Lithium slag powder.

In this experiment, the rubber particles were produced by the rubber processing plant. The size of the rubber particles was 20 mesh, as shown in Fig 1. Lithium slag came from the industrial waste slag-lithium slag abandoned by the factory in Wanzai County, Yichun City, Jiangxi Province, as shown in Fig 2. The components of lithium slag were shown in Table 1. The type of cement was Ordinary Portland cement of conch brand P.O.42.5. The sand included river sand and medium sand. The stone was gravel, and the particle size was 5-20mm. The pouring water was ordinary tap water in the laboratory. The selected steel tube materials were all cold-formed thin-walled processed steel tubes. The test was performed in accordance with GB/ T228-2002 standard [17]. The tensile experiments of steel specimens were performed in the laboratory using a 30t universal testing machine. The experimental value of the yield strength of the steel was 393MPa, and the theoretical value was 401MPa. The experimental values of the elastic modulus and the tensile strength were 2.06 ×  10^5^ MPa and 495MPa, respectively.

**Table 1:** Chemical components of lithium slag.

Composition	SiO_2_	Al_2_O_3_	CaO	SO_3_	TiO_2_	Fe_2_O_3_	K_2_O	MgO	Others
Content (%)	45.9	19.3	9.7	5.97	2.2	1.23	1.2	1.1	13.4

### 2.2 Specimen design

In this paper, concrete test block mix ratio design in accordance with specification for mix proportion design of ordinary concrete [18]. lithium slag and rubber concrete were formulated based on the strength class of C30 ordinary concrete with 30MPa. According to substitution ratios in lithium slag concrete and rubberized concrete[8,19–22], the test was taken to replace the cement and sand in ordinary concrete with lithium slag replacement ratios of 0%, 10%, 20%, and 30% and rubber replacement ratios of 0%, 5%, 10%, 20%, and 30% respectively, and then the base mix proportion was calculated. According to the calculation, the water-cement ratio was 0.46 and the sand ratio was 31%, based on which the dosage of each material was derived and the concrete was formulated to finalize the lithium slag and rubber concrete ratio, which consisted of 11 mix proportions. The size of the circular tube was 89mm in outer diameter, 2mm in thickness, and 267mm in length. Three cubic blocks of 100mm × 100mm × 100mm were made for each ratio for the compressive strength test of the concrete. 11 LSRuCFST stub column specimens were prepared, named by lithium slag replacement ratio and rubber replacement ratio, shown in Table 2.

**Table 2:** Mixing proportion of lithium slag and rubber concrete (kg/m^3^).

Specimens	$\gamma_{LS}/\%$	$\gamma_{R}/\%$	Cement	water	sand	stone	rubber	lithium slag
LS-0-R-0	0	0	2.3756	1.091	2.8796	6.4178	0	0
LS-10-R-0	10	0	2.138	1.091	2.8796	6.4178	0	0.2376
LS-20-R-0	20	0	1.9006	1.091	2.8796	6.4178	0	0.4752
LS-30-R-0	30	0	1.663	1.091	2.8796	6.4178	0	0.7126
LS-20-R-5	20	5	1.9006	1.091	2.7356	6.4178	0.144	0.4752
LS-20-R-10	20	10	1.9006	1.091	2.5916	6.4178	0.288	0.4752
LS-20-R-20	20	20	1.9006	1.091	2.3038	6.4178	0.5758	0.4752
LS-20-R-30	20	30	1.9006	1.091	2.0158	6.4178	0.8638	0.4752
LS-0-R-10	0	10	2.3756	1.091	2.5916	6.4178	0.288	0
LS-10-R-10	10	10	2.138	1.091	2.5916	6.4178	0.288	0.2376
LS-30-R-10	30	10	1.663	1.091	2.5916	6.4178	0.288	0.7126

Note: Specimen number description: LS meant lithium slag, R meant rubber, the first number meant lithium slag powder replacement ratio (percentage), and the second number meant rubber particles replacement ratio (percentage). The following LS-10-R-10 as an example represented the lithium residue replacement ratio of 10% and rubber replacement ratio of 10% specimen.

Before pouring the concrete, the top and bottom sections of the circular steel tubes were ground with a grinding machine until horizontal and smooth to ensure uniform load bearing during the test. The concrete that had been mixed was poured into the tubes and lightly inserted and pounded using an iron bar. When the pouring reaches 4/5 of the tube, the specimen was placed on a vibrating table for vibration. After the vibration was completed, the specimen was moved to the pre-prepared platform, the floating slurry on the upper surface was removed, the water on the surface was dried with a dry cloth, and then the same mix strength cement mortar was used on the platform for leveling, and then covered the top of the specimen with a pressure plate. All poured specimens were placed indoors for maintenance, with watering three times a day for the first week, followed by natural maintenance until 28 days.

### 2.3 Test loading

**Fig 3:**
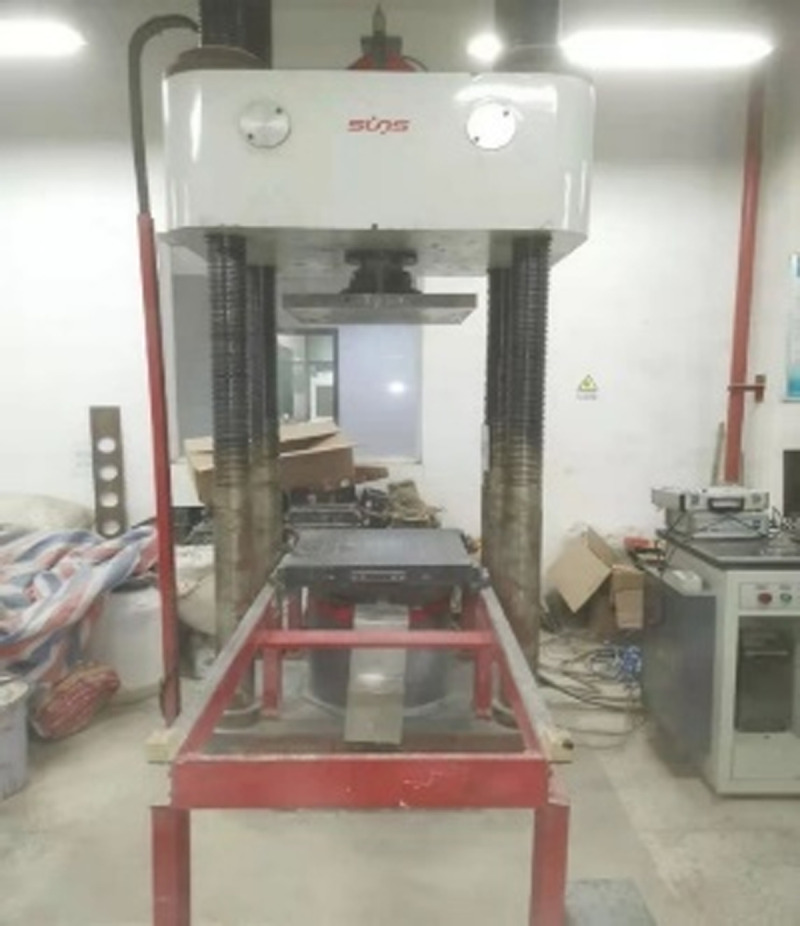
300t pressure testing machine.

**Fig 4:**
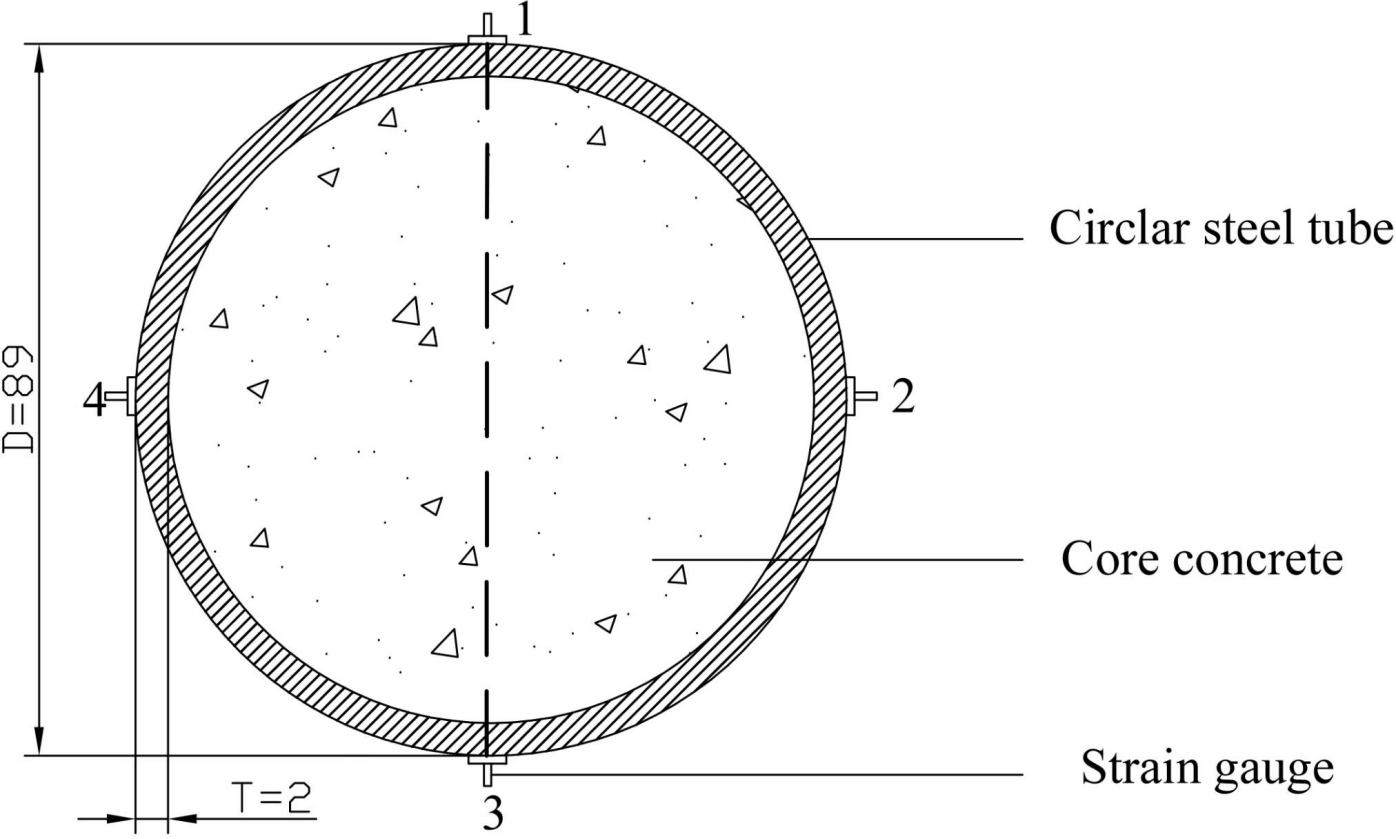
The strain gauge arrangement.

**Fig 5:**
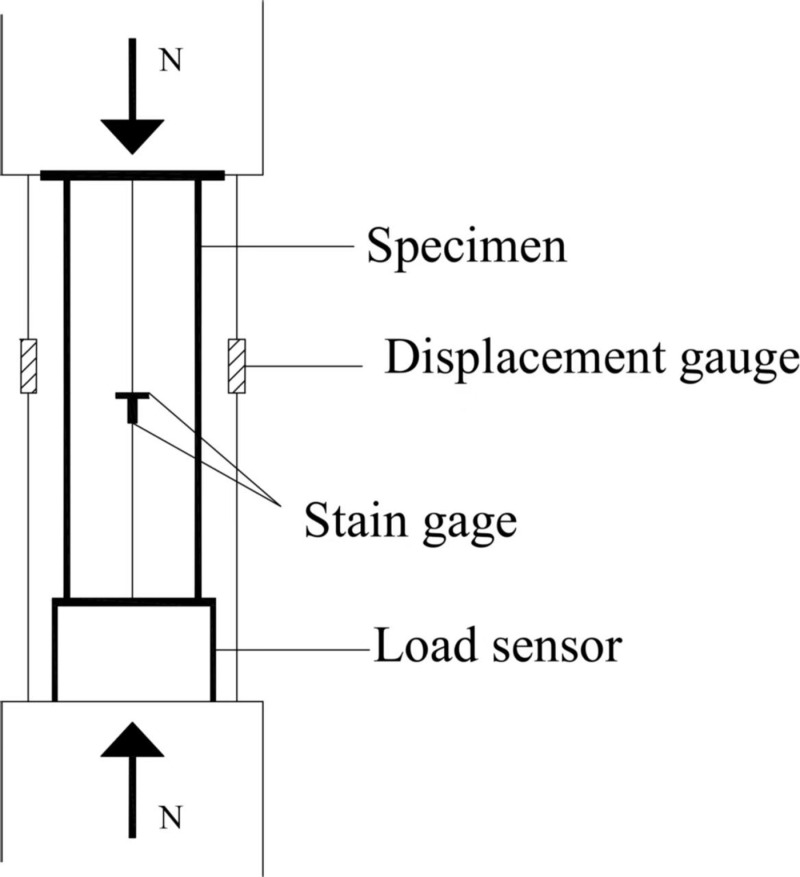
Loading of the specimen.

The axial compression test of the specimen was carried out by a 300t pressure testing machine (as shown from Fig 3). For getting the accurate data, the axial and circumferential strain gauges were applied at 90°intervals on the middle side of the steel tube outer of the specimen (as shown in Fig 4), while two displacement gauges (as shown in Fig 5) were set vertically on the surface of the bottom pressure plate for measuring the axial displacement of the specimen. The speed of loading the specimen was 1.5 mm/min. When the load reduced to about 75% of the maximum load, the test was stopped. If the load decline phase was not significant, the test would be stopped with the axial deformation reached 35mm [23].

## 3. Experimental results and discussions

### 3.1 Experimental phenomena and results

**Fig 6:**
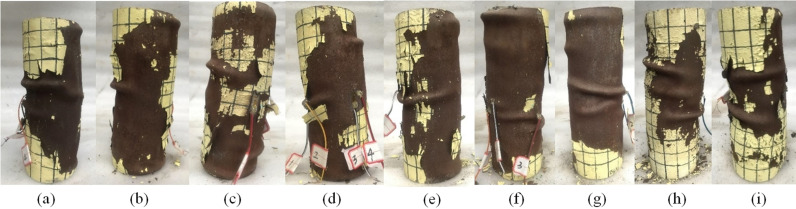
The failure pattern of specimens. (a) LS-0-R-0. (b) LS-10-R-0. (c) LS-20-R-0. (d) LS-30-R-0. (e) LS-20-R-5. (f) LS-20-R-30. (g) LS-0-R-10. (h) LS-10-R-10. (i) LS-30-R-10.

It was found that the partial replacement of cement and fine aggregate by lithium slag powder and rubber particles, respectively, didn’t significantly change the failure modes of CFST columns. And Fig 6 illustrated the typical failure patterns. During the early stage of loading, since the applied load was relatively small, LSRuCFST columns were in the elastic stage, and there were usually no significant changes at the macro level. As the machine continued to load, the concrete in the specimen rubbed against the steel tube inner wall, making a slight ‘ squeak ‘ sound. Local bulges began to appear in the upper, middle, or lower parts of the specimens, illustrating that the specimens were in the phase of elastic-plasticity. The degree of deformation at the bulge continued to increase with the force and slowly expands toward the ends of the specimen. When the maximum load was reached, a crushing sound was heard from the core concrete of the specimen. As loading continued, the axial deformation was increased, and the local bulges became larger. At the same time, there was a certain arc of the bending, and eventually the buckling failure or shear failure occurred [23].

Table 3 showed the experimental results, *f*_*cu*,150_ denoted the compressive strength of concrete cube specimens with a side length of 150 mm, which was obtained by multiplying the strength of concrete cube specimens with a side length of 100 mm by 0.95 [24]. The confinement factor was given by the equation $\xi =\frac{Asfy}{Acfck}$, where *f*_*y*_ represented the yield strength of the steel, $A_{s}$ and $A_{c}$ denoted the cross-sectional areas of steel tube and the core concrete respectively, $f_{ck}$ represented the characteristic value of concrete strength, which was equal to $0.67\cdot f_{cu,150}$ [25]. If the curves of the load-displacement of the specimens didn’t have a significant descending phase or the descending magnitude was small, the ductility factor would not be calculated by selecting the ultimate and yield displacements. The deformation at the yield stage was adopted the definition of strain at the yield stage studied by Ding et al. [25], while the deformation at the failure stage was adopted the axial strain at the ultimate bearing capacity. The axial ductility index was defined by $DI=\varepsilon_{u}/\varepsilon_{0.75}$, where $\varepsilon_{0.75}$ was the longitudinal strain corresponding to a load of 0.75 ∙ $N_{u}$ ($N_{u}$ was the ultimate bearing capacity) before the load reached the ultimate bearing capacity, and $\varepsilon_{u}$ was the longitudinal strain at the ultimate load capacity $N_{u}$As seen from Table 3., when the rubber replacement ratio was zero, except for specimen LS-10-R-0, the ductility coefficients of the specimens were less than the coefficient of CFST columns; with the increase of rubber replacement ratio, the ductility coefficients were higher than the value of CFST columns.

**Table 3:** Test results.

Specimens	$f_{cu,150}$/MPa	$f_{c}$	$N_{u}$/kN	$\varepsilon_{75\%}$	$\varepsilon_{max}$	DI	*ξ*
LS-0-R-0	27.00	21.27	444	0.013	0.039	3.053	2.09
LS-10-R-0	28.89	22.93	480	0.010	0.035	3.607	1.96
LS-20-R-0	31.04	24.83	517	0.015	0.032	2.156	1.82
LS-30-R-0	29.16	23.17	479.1	0.013	0.038	2.896	1.94
LS-20-R-5	26.70	21.01	435	0.016	0.058	3.742	2.12
LS-20-R-10	19.54	14.84	425	0.017	0.053	3.195	2.89
LS-20-R-20	12.62	9.11	368	0.014	0.056	4.013	4.48
LS-20-R-30	9.23	6.41	354	0.019	0.076	4.055	6.12
LS-0-R-10	13.05	9.46	397	0.014	0.048	3.333	4.33
LS-10-R-10	13.72	10.00	411	0.013	0.046	3.550	4.12
LS-30-R-10	14.04	10.26	410.8	0.015	0.055	3.783	4.02

### 3.2 Load displacement curves

**Fig 7:**
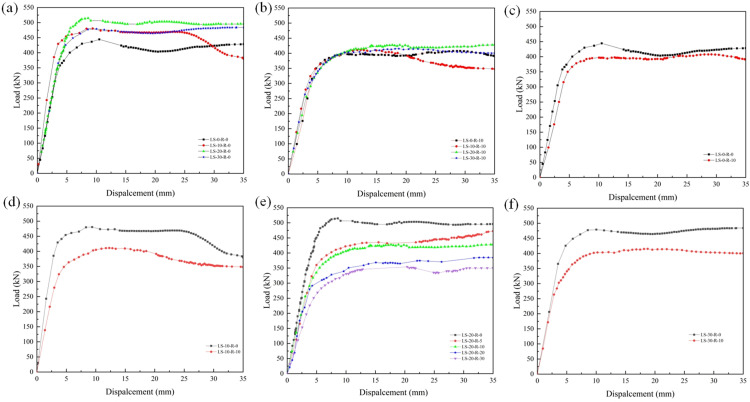
The curves of load-displacement. (a) $\gamma_{R}=0\%$(b) $\gamma_{R}=10\%$(c) $\gamma_{LS}=0\%$(d) $\gamma_{LS}=10\%$(e) $\gamma_{LS}=20\%$(f) $\gamma_{LS}=30\%$.

Fig 7(a), (b) illustrated the curves of the specimen load-displacement with various lithium slag replacement ratios (0, 10%, 20%, and 30%) for 0 and 10% of the rubber replacement ratio, respectively. According to Fig 7(a), it could be concluded that the load-displacement relationship of all the specimens basically varied linearly in the elastic phase with only lithium slag content, and the curves in the elastic phase almost overlapped except for LS-10-R-0, and the slopes of all the curves in the ascending phase didn’t differ much, which indicated that the compressive stiffnesses of the specimens in the phase were not much different. As loading continued, the ultimate bearing capacities of the specimens were in the order of LS-20-R-0, LS-10-R-0, LS-30-R-0, LS-0-R-0. After the load, the bearing capacity of all specimens began to decrease, but due to the radial confinement of the tube, the bearing capacity decreased not much, basically presenting a horizontal state, and only LS-10-R-0 rapidly entered the phase of softening and destruction, indicating that the replacement of the lithium slag would improve the late strength of the specimen. As Fig 7(b) showed, the curves of specimens almost completely overlapped at the 10% rubber replacement ratio. The stiffness of the specimen LS-0-R-10 in the elastic stage was slightly smaller than that of the other three specimens, and its yield strength and ultimate bearing capacity were also slightly smaller than those of the other three specimens. The comparison results showed that the incorporation of lithium slag would have little effect on the properties of the specimens in the case of 10% of the rubber replacement ratio.

Fig 7(c), (f) showed the curves of specimens with various rubber replacement ratios for lithium slag replacement ratios of 0, 10%, 20%, and 30%, respectively. Fig 7(c), (f) showed that the axial compressive stiffness of the elastic phase gradually became smaller with the growth of the rubber replacement ratios; similarly, in the yield phase, the stiffness continued the trend of the elastic phase, and the gap became bigger, while the rubber replacement of the fine aggregate led to the specimens entering into the yield phase in advance; the specimens’ bearing capacities decreased with the growth of the rubber replacement ratios, which indicated that the content would reduce the stiffness of specimens. Thanks to the radial confinement of the steel tube, the bearing capacity of the specimens didn’t decrease much after the ultimate load, except for the specimen LS-10-R-10. The curves showed a flat development, indicating that the specimens had good ductility.

### 3.3 Influence of lithium slag replacement ratios

**Fig 8:**
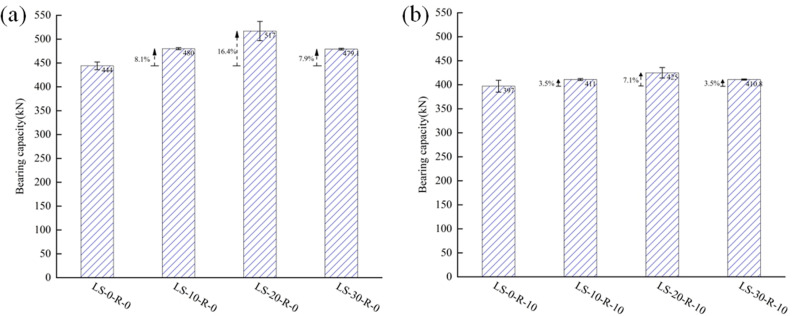
The bearing capacities of specimens of different lithium slag replacement ratios. (a) $\gamma_{R}=0\%$(b) $\gamma_{R}=10\%$.

Figs 8(a)–(b) showed the influences of various lithium slag replacement ratios on the ultimate bearing capacity of the specimens for the case of a fixed value of the rubber replacement ratios. For the case of 0%, compared with the ultimate bearing capacities of LS-0-R-0, those of LS-10-R-0, LS-20-R-0, and LS-30-R-0 were raised by 8.1%, 16.4%, and 7.9%, respectively. In the case of 10%, the ultimate bearing capacities of LS-10-R-10, LS-20-R-10, and LS-30-R-10 were increased by 3.5%, 7.1%, and 3.5%, respectively, compared to that of LS-0-R-10. As a whole, it seemed that the bearing capacities of the specimens were raised and then declined with the growth of the lithium slag replacement ratio. This is due to the fact that LS is a supplementary cementitious material with high volcanic ash properties, which hydrates with the cement and makes the concrete denser, however, over 20%, it was found that some of the LS didn’t hydrate [19–20]. The ultimate bearing capacity of the LSRuCFST column increased by 7.1% with 10% of the rubber replacement ratio and 20% of the lithium slag replacement ratio. The main reason is that the addition of rubber results in an insignificant increase in bearing capacity. In order to improve the range of applications, rubber should be pre-treated [26]. This kind of LSRuCFST stub column could be used in structures with low requirements for bearing capacity.

### 3.4 Influence of rubber replacement ratios

**Fig 9:**
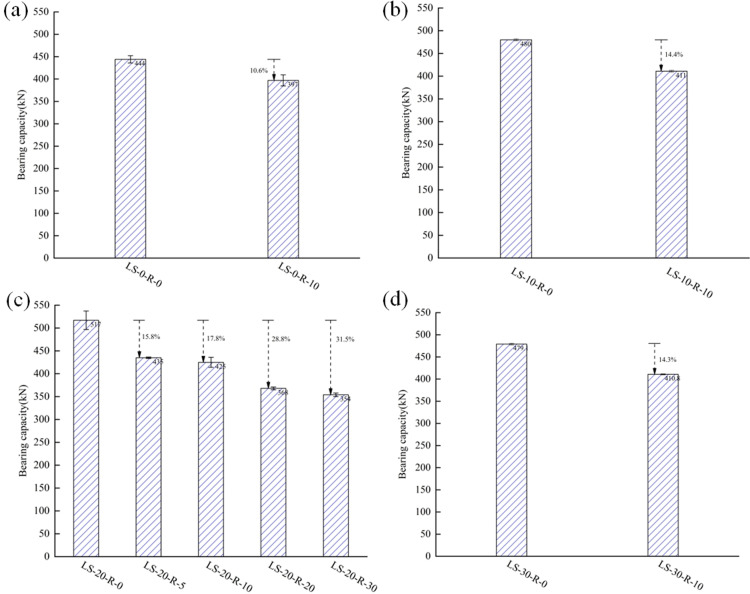
The bearing capacity of specimens of different rubber replacement ratios. (a) $\gamma_{LS}=0\%$(b) $\gamma_{LS}=10\%$(c) $\gamma_{LS}=20\%$(d) $\gamma_{LS}=30\%$.

Fig 9(a), (d) showed the influences of various rubber replacement ratios on the ultimate bearing capacity of the specimens with a constant lithium slag replacement ratio. In the case of 0% lithium slag replacement ratio, the bearing capacity of LS-0-R-10 was decreased by 10.6% compared to that of LS-0-R-0. When the lithium slag replacement ratio was 10%, the bearing capacity of LS-10-R-10 was decreased by 14.4% compared to that of LS-10-R-0. When the lithium slag replacement ratio was 20%, compared with the bearing capacity of LS-20-R-0, the capacities of LS-20-R-10, LS-20-R-20, and LS-20-R-30 were decreased by 17.8%, 28.8%, and 31.5%, respectively. When the lithium slag replacement ratio was 30%, the ultimate bearing capacity of LS-30-R-10 was decreased by 14.3% compared to that of LS-30-R-0. Comparing the data revealed that the replacement ratio of rubber had an important influence on the bearing capacities of the LSRuCFST stub columns. The bearing capacity of specimen decreased by degrees with the replacement ratio [27,28]. Due to the low elastic modulus, smooth texture and lack of adhesion between rubber and mortar, the pores in concrete increase with the increase in admixture, resulting in the decrease of bearing capacity of specimens [29].

## 4. Calculation of bearing capacity

### 4.1 ACI (2005) [30]

For axial compression of the stub columns, the bearing capacity of CFST stub columns was obtained by the following formula.


N1=0.85Φ(Asfy+0.85fcAc) (1)

Where Φ denoted the discount factor, taking the value 0.75; *f*_*y*_ denoted the yield strength of the steel;$f_{c}^{'}$denoted the compressive strength of the cylindrical concrete specimen, obtained with the model of L’Hermite [31]:


$$f^{\prime }c=(0.76+0.2\log10(\frac{fcu}{19.6}))fcu$$ (2)

### 4.2 AIJ(1997)[32]

For the stub columns, the bearing capacity of CFST could be obtained according to the following equation:


$$N_{2}=1.27AsF+0.85f^{\prime }cAc$$ (3)

Where F denoted the standard value of the steel strength, $F=\text{min}\left(f_{y},0.7f_{u}\right)$$f_{u}$ for the tensile strength of steel.

### 4.3 AISC(2005)[33]

The bearing capacity of CFST could be obtained according to the following equations:


$$N3= \{\begin{array}{c} \varphi \left[0.658^{\frac{N0}{Ncr}}\right]N0(Ncr\geq 0.44N0)\\ 0.877\varphi Ncr(Ncr<0.44N0) \end{array}$$ (4)

$$N0=Asfy+C1f^{\prime }cAc$$ (5)

$$Ncr=\frac{\pi^{2}}{{\left(KL\right)}^{2}}(EIeff)$$ (6)

Where *φ* was the discount factor, taking the value 0.75; $C_{1}$ was the coefficient, $C_{1}=0.95$ for circular CFST; $C_{1}=0.85$ for square and rectangular CFST; L was the length of the component used for the calculation; K denoted the effective length factor.

$EI_{eff}$ was the equivalent stiffness of CFST, calculated as follows:


EIeff=EsIs+C2EcIc (7)

Where $E_{s}$$E_{c}$ were the elastic modulus of steel and concrete, respectively; $C_{2}$ was the coefficient, $C_{2}=0.6+2\alpha \leq 0.9$, which *α* was equal to $A_{s}/\left(A_{s}+A_{c}\right)$.

### 4.4 BS5400(2005) [34]

For the circular CFST, considering that the strength of core concrete would increase in the case of triaxial compression, the bearing capacity of the CFST stub column was calculated by the following equation:


$$N4=0.95Asf^{\prime }y+0.45Acfcc$$ (8)

Where$f_{cc}$was the compressive strength of core concrete for triaxial compression, determined according to the following formula:


$$fcc=fcu+C1\cdot fy\cdot \frac{t}{D}$$ (9)

$f_{y}^{'}$denoted the yield strength of the steel after discounting, determined by the following formula:


$$f^{\prime }y=C2\cdot fy$$ (10)

$f_{cu}$was the compressive strength of a concrete cube cured for 28 days;

*t* denoted the thickness of steel tube; *D* was the external diameter of the tube;

$C_{1}$, $C_{2}$ were the calculated factors, as determined in Table 4.

**Table 4:** The values of the calculation coefficients $C_{1}$, $C_{2}$_._

$l_{e}/D^{*}$	$C_{1}$	$C_{2}$
0	9.47	0.76
5	6.4	0.8
10	3.81	0.85
15	1.8	0.9
20	0.48	0.95
25	0	1

### 4.5 GB50936-2014 [35]

For stub columns, the bearing capacity of CFST could be obtained according to the following equation:


$$N_{5}=(1.212+B_{1}\xi +C_{1}\xi^{2})\cdot f_{c}\cdot A_{sc}$$ (11)

Where *B*_1_ and $C_{1}$were coefficients, calculated as follows:


$$B1=0.1759\cdot \frac{fy}{235}+0.974$$ (12)

$$C1=-0.1038\cdot \frac{fck}{20}+0.0309$$

ξ was the characteristic value of the confinement coefficient of a component section, equal to $\frac{ASfy}{Acfck}$;

### 4.6 EC4(2004) [36]

For CFST, in the case of the relative slenderness ratio $\bar{\lambda }\leq 0.5$ and the load eccentricity e≤D/10, since the confinement influence of the tube on the core concrete should be considered, the bearing capacity should be obtained according to the following equation:


$$N\text{6}=\eta s\frac{fy}{\gamma s}\cdot As+\left(1+\eta c\cdot \frac{t}{D}\cdot \frac{fy}{f^{\prime }c}\right)\cdot \frac{f^{\prime }c}{\gamma c}\cdot Ac$$ (13)

Where $\gamma_{s}$ denoted the material partial coefficient of steel, and its value was 1.0; $\gamma_{s}$ was the material partial coefficient of concrete, and its value was 1.5.

When the eccentric distance e=0$\eta_{s}=\eta_{s0}$$\eta_{c}=\eta_{c0}$where the calculation formulas of $\eta_{s0}$ and $\eta_{c0}$ were as follows:


$$\eta s0=0.25\cdot \left(3+2\bar{\lambda }\right)\left(\eta s0\leq 1\right)$$ (14)

$$\eta c0=4.9-18.5\bar{\lambda }+17{\bar{\lambda }}^{2}\left(\eta c0\geq 0\right)$$ (15)

**Fig 10:**
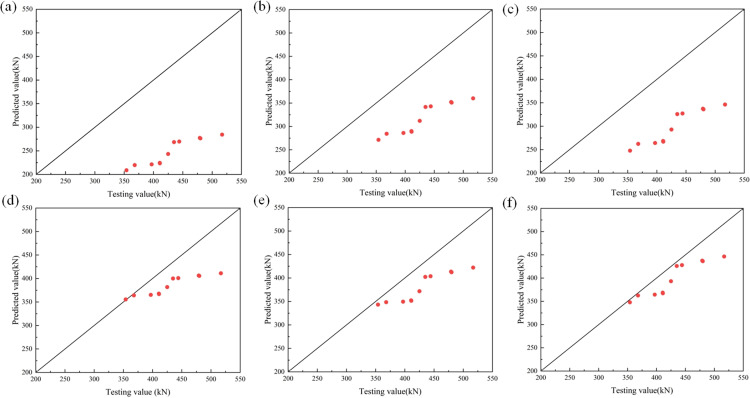
Comparison between test value and predicted value. (a) ACI2005. (b) AIJ1997. (c) AISC2005. (d) BS5400. (e) GB50936-2014. (f) EC4(2004).

Table 5 gave the results of the calculated bearing capacity for each code, and Table 6 gave the comparisons between the calculated bearing capacity and the test values for each code. From Table 6 and Figs 10(a)–(f), it could be found that the results of these six codes were conservative, and the results of EC4(2004) were relatively close to the test results.

**Table 5:** Theoretical and testing values of compressive bearing capacity.

Specimens	N_test_	N_1_	N_2_	N_3_	N_4_	N_5_	N_6_
LS-0-R-0	444	269.68	342.97	327.21	400.77	403.67	427.81
LS-10-R-0	480	276.47	350.97	336.05	405.59	412.21	436.22
LS-20-R-0	517	284.26	360.13	346.18	411.08	422.08	446.34
LS-30-R-0	479.1	277.45	352.12	337.32	406.28	413.44	437.49
LS-20-R-5	435	268.60	341.71	325.81	400.00	402.33	426.00
LS-20-R-10	425	243.34	311.99	292.92	381.73	371.82	393.16
LS-20-R-20	368	219.84	284.34	262.29	364.07	348.43	362.45
LS-20-R-30	354	208.78	271.34	247.87	355.41	343.28	347.85
LS-0-R-10	397	221.26	286.02	264.16	365.16	349.53	364.32
LS-10-R-10	411	223.50	288.65	267.07	366.87	351.37	367.25
LS-30-R-10	410.8	224.57	289.91	268.46	367.69	352.30	368.66

**Table 6:** The comparison of design and testing values of the bearing capacity.

Specimens	N_1_/N_test_	N_2_/N_test_	N_3_/N_test_	N_4_/N_test_	N_5_/N_test_	N_6_/N_test_	N_new_/ N_test_
LS-0-R-0	0.61	0.77	0.74	0.90	0.91	0.96	1.02
LS-10-R-0	0.58	0.73	0.70	0.84	0.86	0.91	0.99
LS-20-R-0	0.55	0.70	0.67	0.80	0.82	0.86	0.96
LS-30-R-0	0.58	0.73	0.70	0.85	0.86	0.91	0.99
LS-20-R-5	0.62	0.79	0.75	0.92	0.92	0.98	1.07
LS-20-R-10	0.57	0.73	0.69	0.90	0.87	0.93	1.00
LS-20-R-20	0.60	0.77	0.71	0.99	0.95	0.98	1.03
LS-20-R-30	0.59	0.77	0.70	1.00	0.97	0.98	0.99
LS-0-R-10	0.56	0.72	0.67	0.92	0.88	0.92	0.99
LS-10-R-10	0.54	0.70	0.65	0.89	0.85	0.89	0.97
LS-30-R-10	0.55	0.71	0.65	0.90	0.86	0.90	0.96
Mean	**0.576**	**0.738**	**0.694**	**0.901**	**0.887**	**0.930**	**0.998**
Std. Dev	**0.025**	**0.031**	**0.032**	**0.060**	**0.046**	**0.041**	**0.032**

### 4.7 Improvement of the bearing capacity formula

Based on the comparison results of the above table, the calculation results were corrected based on the EC4 (2004) specification. Because both lithium slag and rubber particles had an impact on the compressive strength of concrete specimens, which affected the confinement coefficient of CFST, it could modify the bearing capacity of the concrete part of the formula by using a quadratic function form, which could be expressed as:


$$Ncu=\eta s\frac{fy}{\gamma s}\cdot As+\left(1+(A+B\cdot R+C\cdot L+D\cdot R^{2}+E\cdot L^{2}+F\cdot R\cdot L)\eta c\cdot \frac{t}{D}\cdot \frac{fy}{f^{\prime }c}\right)\cdot \frac{f^{\prime }c}{\gamma c}\cdot Ac$$ (16)

Where R and L were rubber and lithium slag replacement ratios respectively. Using linear regression, the results could be obtained *A* = 1.18611, *B* = 0.01308, *C* = 0.00228, *D* = -1.91674 × 10^-4^, *E* = 9.7663 × 10^-5^, *F* = -8.328 × 10^-4^.

**Fig 11:**
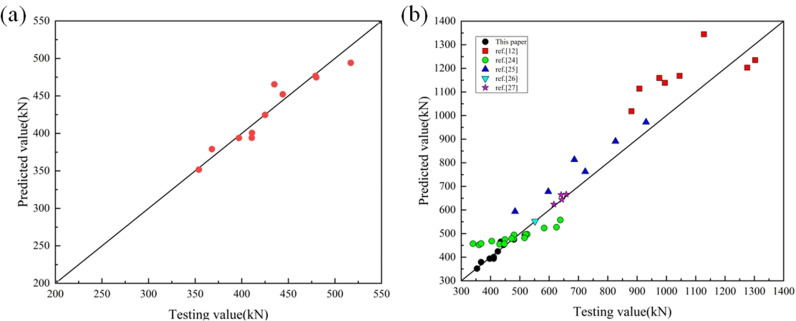
Comparison of the results of modified equation and test. (a) This paper. (b) Others papers.

With the modified formula, it could be obtained that the mean and the variance were 0.998 and 0.07, respectively. It could be concluded that the modified results were consistent with the testing ones in Fig 11(a).

To verify whether the modified formula was reasonable, the specimens obtained in Reference [15,37–40] were calculated. The comparison results were shown in Table 7 and Fig 11(b). It showed that the modified equation could be employed to calculate the bearing capacity of the LSRuCFST stub columns.

**Table 7:** The design parameters and bearing capacities of specimens.

Ref.	Specimens	D/mm	L/mm	t/mm	$\gamma_{R}/\%$	$\gamma_{LS}/\%$	$f_{C}^{'}$/MPa	Fy/MPa	N_test_/kN	N_new_/kN	N_new_/N_test_
This paper	LS-0-R-0	89	267	2.00	0	0	21.271	393.0	444.0	452.2	1.02
	LS-10-R-0	89	267	2.00	0	10	22.930	393.0	480.0	474.8	0.99
	LS-20-R-0	89	267	2.00	0	20	24.830	393.0	517.0	494.2	0.96
	LS-30-R-0	89	267	2.00	0	30	23.168	393.0	479.1	476.6	0.99
	LS-20-R-105	89	267	2.00	5	20	21.009	393.0	435.0	465.4	1.07
	LS-20-R-10	89	267	2.00	10	20	14.844	393.0	425.0	424.7	1.00
	LS-20-R-20	89	267	2.00	20	20	9.109	393.0	368.0	379.0	1.03
	LS-20-R-30	89	267	2.00	30	20	6.411	393.0	354.0	351.6	0.99
	LS-0-R-10	89	267	2.00	10	0	9.457	393.0	397.0	393.9	0.99
	LS-10-R-10	89	267	2.00	10	10	10.002	393.0	411.0	400.6	0.97
	LS-30-R-10	89	267	2.00	10	30	10.264	393.0	410.8	394.1	0.96
Ref. [37]	C-1	114	456	2.00	0	0	18.140	382.0	638.0	557.9	0.87
	C-2	114	456	2.00	2.5	0	14.470	382.0	625.0	526.8	0.84
	C-3	114	456	2.00	5	0	13.970	382.0	583.0	523.5	0.90
	C-4	114	456	2.00	10	0	10.640	382.0	525.0	497.2	0.95
	C-5	114	456	2.00	20	0	7.250	382.0	449.0	474.9	1.06
	C-6	114	456	2.00	30	0	5.300	382.0	404.0	468.0	1.16
	C-7	114	456	2.00	2.5	0	11.050	382.0	520.0	497.0	0.96
	C-8	114	456	2.00	5	0	9.170	382.0	516.0	481.7	0.93
	C-9	114	456	2.00	10	0	8.450	382.0	479.0	478.0	1.00
	C-10	114	456	2.00	20	0	5.000	382.0	431.0	455.0	1.06
	C-11	114	456	2.00	30	0	3.570	382.0	361.0	452.4	1.25
	C-12	114	456	2.00	2.5	0	10.720	382.0	480.0	494.4	1.03
	C-13	114	456	2.00	5	0	8.850	382.0	472.0	478.8	1.01
	C-14	114	456	2.00	10	0	6.000	382.0	447.0	456.4	1.02
	C-15	114	456	2.00	20	0	5.380	382.0	368.0	458.3	1.25
	C-16	114	456	2.00	30	0	4.090	382.0	340.0	457.1	1.34
Ref. [38]	C114X3_235_0	114	300	2.70	0	0	38.180	270.2	723.1	762.4	1.05
	C114X3_235_5	114	300	2.70	5	0	28.180	270.2	597.0	677.6	1.14
	C114X3_235_15	114	300	2.70	15	0	17.630	270.2	483.7	593.7	1.23
	C114X3_275_0	114	300	3.20	0	0	38.180	358.9	930.7	971.8	1.04
	C114X3_275_5	114	300	3.20	5	0	28.180	358.9	826.3	890.9	1.08
	C114X3_275_15	114	300	3.20	15	0	17.630	358.9	686.0	813.0	1.19
Ref. [39]	T20SF0R10t2	86	188	2.00	20	0	54.270	342.0	551.8	551.9	1.00
Ref. [40]	C1	89	300	3.00	0	0	21.271	426.0	616.0	624.0	1.01
	C2	89	300	3.00	0	10	22.930	426.0	643.0	643.8	1.00
	C3	89	300	3.00	0	20	24.830	426.0	658.5	666.1	1.01
	C4	89	300	3.00	0	30	23.168	426.0	640.6	664.3	1.04
Ref. [15]	N4	150	450	3.55	0	0	25.320	326.0	1303.0	1234.8	0.95
	N4-1-10	150	450	3.55	10	0	22.440	326.0	1276.0	1202.9	0.94
	N4-1-20	150	450	3.55	20	0	16.890	326.0	995.0	1138.4	1.14
	N4-1-30	150	450	3.55	30	0	16.640	326.0	976.0	1159.1	1.19
	N3-1-20	150	450	2.50	20	0	16.890	394.0	881.0	1018.4	1.16
	N5-1-20	150	450	4.50	20	0	16.890	326.0	1128.0	1344.2	1.19
	N4-3-20	150	450	3.55	20	0	18.900	326.0	1045.0	1168.1	1.12
	N4-0.5-20	150	450	3.55	20	0	15.240	326.0	908.0	1114.0	1.23

## 5. Conclusion

1) The axial compression failure patterns of LSRuCFST stub column were similar to these of ordinary CFST stub column, and the failure patterns were local buckling or shear damage.
2) At the same rubber replacement ratio, the bearing capacity of the specimen was the largest for 20% of the lithium slag replacement ratio. When the rubber replacement ratio was 10%, and the lithium slag replacement ratio was 10% -30%, the bearing capacity could be increased by 3.5% -7.1%.
3) Under the same lithium slag replacement ratio, the stiffness, the yield strength, and the ultimate bearing capacity of the specimens decreased with the raise of the rubber replacement ratio, while the ductility of the specimen increased. With 20% of the lithium slag replacement rate and 10% of the rubber substitution rate, the bearing capacity is optimal and the ductility is good. In order to improve the range of applications, the mechanical properties after the addition of pretreated rubber can be investigated in future tests.
4) The predicted results of the EC4 (2004) were relatively better. However, in order to fully utilize the material properties, the replacement ratios of lithium slag and rubber were used to modify the code in this paper. The results of the modified formula were compared with the testing ones, and the error was within 7%.

In order to analyze the characteristics of LSRuCFST in detail, the researches of mechanical properties under different loads, seismic performance and bonding performance should be carried out in the future work.

## Supporting information

S1 Data D1. (XLSX)
